# Patient Satisfaction Using Breast-Q After Breast Reconstruction in Male-to-Female Transgender Patients

**DOI:** 10.1093/asjof/ojad027.011

**Published:** 2023-04-14

**Authors:** Haley Desjardins, Alec McCranie, David Mathes, Corinne Wong

**Affiliations:** University of Colorado, Aurora, CO; University of Colorado, Aurora, CO; University of Colorado Anschutz Medical Campus, Aurora; University of Colorado, Aurora, CO; Denver Health Medical Center, Denver, CO

## Abstract

**Goals/Purpose:**

Male-to-female top surgery comprises an increasing portion of breast augmentations performed by plastic surgeons across the nation. Many studies have been performed in cis-females reporting improved overall satisfaction with their breasts, as well as improved psychosocial, sexual, and physical well-being utilizing the validated BREAST-Q questionnaire. Satisfaction in this transgender population has not been well studied using the current standard of care in the United States, non-textured implants. The authors conducted a retrospective study following male-to-female patients who received non-textured implants as part of their breast reconstruction.

**Methods/Technique:**

The authors searched electronic medical records for patients aged 18 years or older who underwent breast augmentation for male-to-female top surgery at Denver Health Medical Center between 2018 and 2021. Survey evaluation using the validated BREAST-Q questionnaire was administered via telephone or in clinic at 6 months and 1 year after breast implant placement. Breast-Q scores for psychosocial, sexual, physical well-being, satisfaction with breasts, and overall post-satisfaction subscales were collected and converted to the equivalent Rasch transformed score. Because this was a retrospective study, median pre-operative subscale scores were utilized from another study that looked at satisfaction in the use of anatomical textured implants in transgender patients by Weigert et al. These pre-operative median scores were compared with patient scores at 6 and 12 months post-operatively.

**Results/Complications:**

30 male-to-female transgender patients were contacted to participate in this study. 24 patients volunteered to participate, and 6 declined. BREAST-Q survey results demonstrate significantly improved satisfaction post-operatively at 6 and 12 months when compared to median pre-operative scores for psychosocial (p <0.001; p <0.001), sexual (p<0.001; p <0.001), physical well-being (p<0.001; p <0.001), and satisfaction with breasts (p<0.001; p <0.001). Results demonstrate that some subscales of post-operative satisfaction increased with more time from surgery. Between 6 and 12 months post-operatively, patients experienced significant improvement in physical (p<0.001), sexual (p<0.007), and psychosocial well-being (p<0.019). No significant change was seen in satisfaction with outcome or satisfaction with breast between 6 and 12 months.

**Conclusion:**

Male-to-female transgender patients exhibit a significant increase in satisfaction following breast augmentation when assessed using the validated BREAST-Q questionnaire. Counseling this patient population pre-operatively, providers can use these results to better advise patients and set reasonable post-operative expectations. Further studies investigating long-term satisfaction in larger cohorts are needed.

